# A Joint Matrix Completion and Filtering Model for Influenza Serological Data Integration

**DOI:** 10.1371/journal.pone.0069842

**Published:** 2013-07-25

**Authors:** Xiao-Tong Yuan, Tong Zhang, Xiu-Feng Wan

**Affiliations:** 1 School of Information and Control, Nanjing University of Information Science and Technology, Nanjing, Jiangsu, China; 2 Department of Statistics, Rutgers University, Piscataway, New Jersey, United States of America; 3 Department of Basic Sciences, Mississippi State University, Mississippi State, Mississippi, United States of America; University of Georgia, United States of America

## Abstract

Antigenic characterization based on serological data, such as Hemagglutination Inhibition (HI) assay, is one of the routine procedures for influenza vaccine strain selection. In many cases, it would be impossible to measure all pairwise antigenic correlations between testing antigens and reference antisera in each individual experiment. Thus, we have to combine and integrate the HI tables from a number of individual experiments. Measurements from different experiments may be inconsistent due to different experimental conditions. Consequently we will observe a matrix with missing data and possibly inconsistent measurements. In this paper, we develop a new mathematical model, which we refer to as Joint Matrix Completion and Filtering, for HI data integration. In this approach, we simultaneously handle the incompleteness and uncertainty of observations by assuming that the underlying merged HI data matrix has low rank, as well as carefully modeling different levels of noises in each individual table. An efficient blockwise coordinate descent procedure is developed for optimization. The performance of our approach is validated on synthetic and real influenza datasets. The proposed joint matrix completion and filtering model can be adapted as a general model for biological data integration, targeting data noises and missing values within and across experiments.

## Introduction

Influenza virus causes both seasonal epidemics and pandemics, and continues to present a threat to public health. Antigenic changes by drift or shift at influenza surface glycoproteins, especially hemagglutinin, changes its antigenic properties, and thus allows influenza virus to evade the accumulating herd immunity from influenza infection or vaccination [1], [2]. Serological assays such as Hemagglutination Inhibition (HI) and Micro Neutralization (MN) are routine procedures used in antigenic variant identification [3].

A serological data can be viewed as an 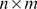 matrix, where 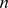 and 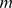 are the numbers of antigens and antisera in the assays, respectively. This matrix is used to quantify (direct or indirect) binding reactions between the two comparing antigen and antiserum. Each matrix entry (titre) can be a numeric value (high reactor), or a low reactor, or a missing value [4]. The low reactors are results of the detection limit of a serological assay, marked as 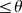, where 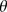 is a threshold indicating the detection limit. The missing values are generally caused by the limitation of resources.

In many cases, it would not be possible to perform all pairwise comparisons between antigen and antiserum in each individual experiment. Thus, we have to combine and integrate the datasets from a number of individual experiments, each of which could be a separate dataset (matrix with missing values) by itself. Measurements from different experiments may be inconsistent due to different experimental conditions. Consequently we will observe a matrix with missing data and possibly inconsistent measurements. Taking the HI assay as an example, typically less than 15 reference antisera (antibodies) are used in each assay but the number of test antigens (viruses) can be more than 100. It is not possible to perform HI for all pairs of antigen and antiserum reactions. Thus, the resulting HI table is generally incomplete. The data absence in these low throughput biological experiments could be up to 95%.

Integration of influenza serological data is critical for vaccine strain selection and pandemic preparedness by providing an antigenic “bluemap” for influenza viruses, including contemporary and historical human influenza viruses and zoonotic influenza viruses. However, integration of influenza HI datasets is not trivial because HI data are notoriously noisy within and across the experiments. A number of factors can affect the robustness of HI assays [3]:

- Reference antisera. The antisera batch (from different challenge experiments), storage conditions (temperature time), and frozen-thawing can affect HI titre values.
- Types and batches of red blood cells. The red blood cells from different animal species affect the HI titre values dramatically, and the red blood cells from the same species can affect the HI titre values.
- Variations in supplies. The types of plates (e.g., “U” or “V” shape) can affect the HI data interpretation.
- Error from personnel.

Among these parameters, the antisera and red blood cells will probably most significantly affect the results from HI assays [3]. To date, there is still lack of robust computational models for integrating influenza HI data.

This paper proposes a new mathematical model called Joint Matrix Completion and Filtering Model, for influenza serological data integration. We address the major challenges caused by the incompleteness and uncertainty in the observation. The proposed model simultaneously handles these two challenges by assuming that the underlying joint table is low rank and carefully modeling different levels of random effects in each individual data table. We develop an efficient blockwise coordinate descent procedure for optimization. The performance of our model is validated on synthetic and real influenza datasets.

### Joint Matrix Completion and Filtering Model for Biological Data Integration

In this study we focus on influenza HI data. There are two major challenges in HI table integration: (i) to complete missing entries; (ii) to handle noisy observations. We propose a joint matrix completion and filtering method to simultaneously address these two challenges. The basic idea of this model is to approximate the merged data matrix using low-rank matrix decomposition, and to incorporate the experimental bias of individual tables. The challenge of missing data is handled by the low-rank matrix model, where we have assumed that the antigens and antisera can be represented by latent low-dimensional vectors, and the pairwise inner product of these vectors approximate HI titre values (including missing entries). The low-rank assumption of the merged HI table is motivated from the intuition that its rows corresponding to those antiviruses with similar antigenic characterization are expected to be linearly dependent. Such a low-rank assumption has also been made in our previous work [4] which was shown to work quite well in completing a manually merged HI table from [5]. The challenge of noise is handed by a two-level hierarchical model which incorporates the biases of antisera in different experiments and the system noise. The antiserum-level bias should be considered because the antisera batch (from different challenge experiments), storage conditions (temperature time), and frozen-thawing can affect HI titre values. The system noise comes from a number of sources such as types and batches of red blood cells, variations in supplies, and error from personnel.

By combining the above two ideas, the joint model can be mathematically posed as follows:
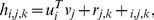
(1)where 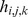 are the observed HI titre values between the antigen-antiserum pairs 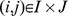 in table 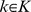, 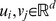 are latent representing vectors for antigen 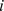 and antiserum 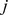, respectively, 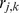 are the (deterministic) bias of antiserum 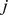 in table 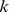, and 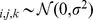 are the i.i.d. Gaussian random noises with unknown variance 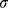. Our goal is to estimate the parameters 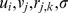 from the observations 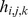.

We propose to estimate the parameters via solving the maximum log-likelihood objective which is equivalent to the following minimization problem:
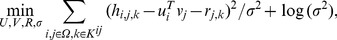
(2)where 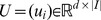, 
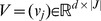
, and 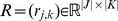. The summation of 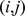 is taken over 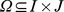 which is the set of antigen-antiserum pairs observed in at least one table. The summation of 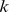 is taken over 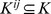 which is the set of tables in which the HI titre between antigen 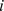 and antiserum 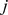 are observed. Note that the estimation of 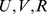 is separated from that of 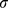 in model (2). That is, we may first estimate 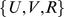 via the following least square formulation



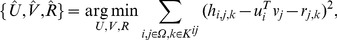
(3)and then estimate 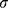 according to
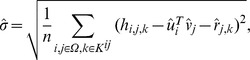
where 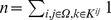 is the total number of observations in all HI tables.

We present a blockwise coordinate descent procedure to solve the problem (3). Starting from an initialization 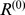, the algorithm alternates between solving the following two subproblems on 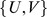 and on 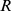 respectively:
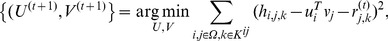
(4)

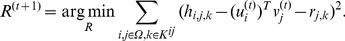
(5)


In our implementation, we initialize the algorithm with 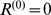 which works satisfactory in the empirical study. We next describe how to solve the subproblems (4) and (5).

Let us consider the minimization of subproblem (4), which is equivalent to the following problem of rank-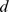 matrix factorization with missing values:
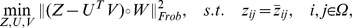
where 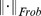 denotes the Frobenius norm of matrix, 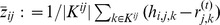, 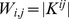 if 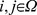 and 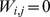 otherwise. This problem is well studied in the matrix completion literature and a number of efficient algorithms are available online [6]–[10]. Here we utilize the *LMaFit* solver developed by [6] which works well for our problem.

Concerning the minimization problem (5), it is not difficult to check that 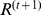 has the following closed-form solution: for each 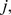


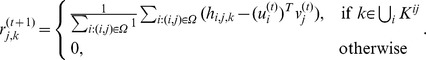



After recovering the merged HI table, we will project the influenza antigens onto a two-dimensional antigenic cartography. Antigenic cartography presents an intuitive way to validate our computational models with biological domain knowledge. The details can be found in the Methods and Materials section.

## Results

### Parameter Selection

There is one free parameter, the rank 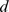 for matrix completion, in our model. For the simulated data and the influenza HI data of 2009 H1N1 an H3N2 used in this study, a 10-fold cross validation suggested a value of 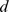 between 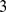 and 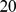 for achieving low Root Mean Squared Error (RMSE). For model initialization, we simply use the mean table as the starting point. We have also tried random initialization in the vicinity of the mean table. The unreported experimental results suggest that the solution of our model is insensitive to these initialization schemes.

### Data Integration on Simulated HI Dataset

To demonstrate the effectiveness of our model in influenza data integration, we performed Monte Carlo simulation using 100 randomly generated HI tables according to the joint model (1) (see Materials and Methods section). The ground truth full HI table contains 600 antigens versus 100 antisera and its rank is 10. We further respectively group antigens and antisera into 10 groups. The intra group HI titre is set to be high while the inter group HI titre is set to be low. Six noise level configurations 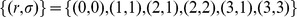 are considered in the simulation.

**Figure 1:**
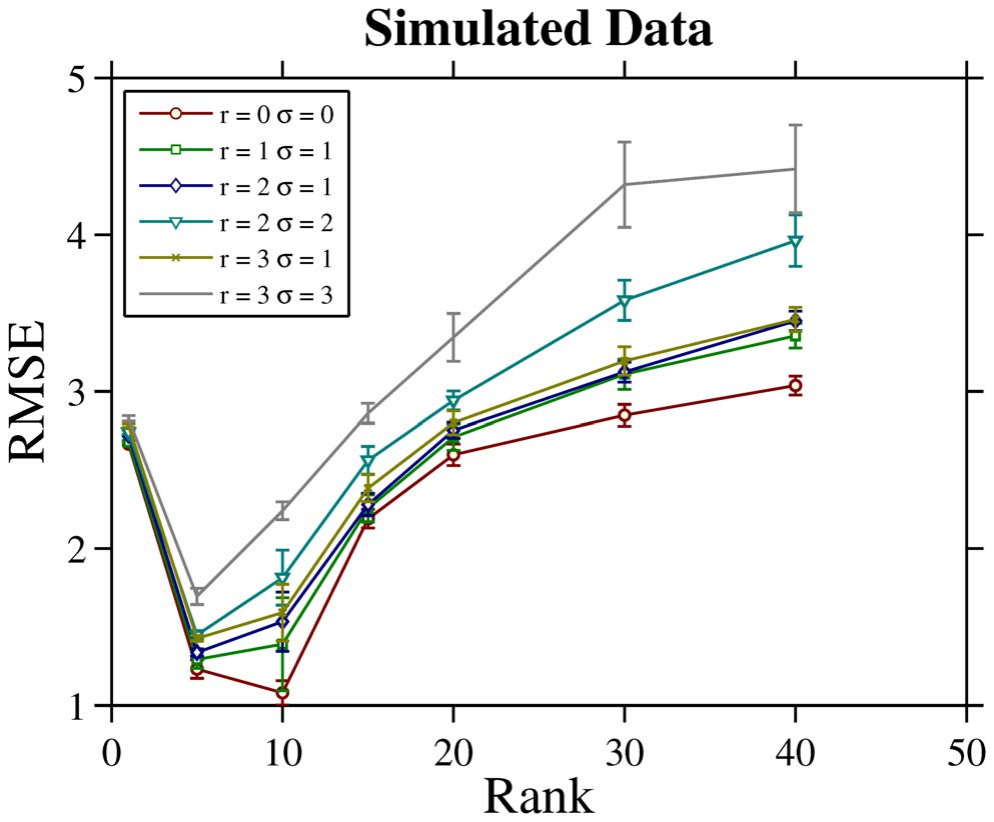
RMSE versus rank curves achieved by our method on the simulated data. It can be observed that when data is noise free, the minimal RMSE is achieved at the ground truth rank 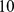. For noisy data, the minimum is attained at rank 5.

We use RMSE between the recovered HI titre and the true HI titre as a measure of accuracy. We repeat the experiment 10 times, and report the mean and standard deviation of RMSE. Figure 1 shows the RMSE versus rank curves obtained under different noise level configurations. From this group of curves we can make the following observations: (i) RMSE increases as the noise level increases, especially when rank is relatively large; (ii) the lowest RMSE is achieved at the rank close to ground truth rank 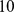; and (iii) the lowest RMSE is insensitive to noise level. The second and third observations confirm that low-rank approximation and hierarchical noise model are effective for accurately and robustly imputing the missing entries.

**Figure 2:**
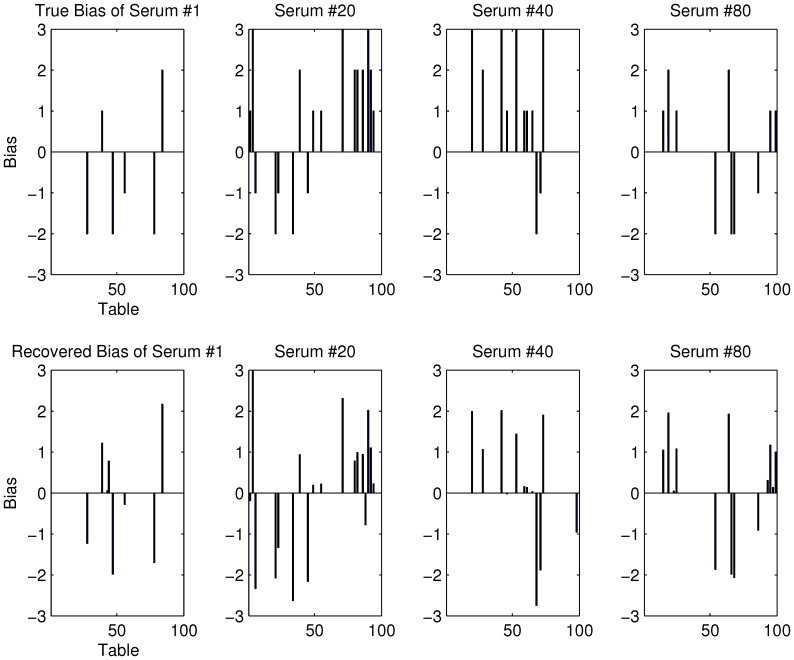
Antiserum bias distribution. Top row: the ground truth antiserum bias added to the simulated tables for antisera #1, #20, #40, #80. Bottom row: the recovered antiserum bias by our algorithm for the same four antisera. The noise level configuration for this group of experiment is 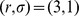. The rank value 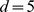 is selected in our model.

We now compare the proposed model to a baseline method which simply averages the measurements observed at each entry and then perform matrix completion over the resultant mean table. We call this method *average model*. This can be viewed as a simplified model of ours by setting 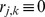 in Equation (2) (see Materials and Methods section). Table 1 lists in rows 2 and row 3 the RMSE achieved by these two methods respectively. Note that the only difference between our model and the average model is whether or not the antiserum bias is explicitly modeled. These results clearly showed that the explicit estimation of the antiserum bias improves the matrix completion accuracy. Figure 2 shows the ground truth antiserum biases we add to the data and the recovered biases 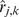 by our model with the noise level configuration 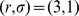. The rank value parameter is set to be 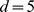 which is optimal according to Figure 1. We can see from this qualitative result that the recovered antiserum bias is close to the ground truth in terms of bias distribution and direction. The RMSE values between the estimated antiserum bias and the ground truth antiserum bias are listed in the row 4 and row 5 of Table 1, respectively for our model and the average model. Here the RMSE of antiserum bias by the average model is estimated in a similar way as in our model (recall that the average model can be taken as a special case of our model). This group of quantitative comparisons confirm the advantage of our model over the average model in recovering antiserum bias.

**Table 1:** Simulated HI dataset: RMSE of HI titre and antiserum bias under different noise level configurations.

Measurements	Methods	(*r*,*σ*) = (1,1)	(2,1)	(2,2)	(3,3)
RMSE	Our Model	1.213 (0.049)	1.336 (0.043)	1.414 (0.066)	1.624 (0.061)
(*in HI titre*)	Average Model	1.228 (0.065)	1.602 (0.052)	1.663 (0.058)	2.004 (0.068)
RMSE	Our Model	1.054 (0.022)	0.984 (0.016)	1.005 (0.013)	1.091 (0.026)
(*in antiserum bias*)	Average Model	1.078 (0.026)	1.225 (0.031)	1.225 (0.028)	1.403 (0.035)

These figures are generated by setting rank values as the optimal ones according to Figure 1.

**Figure 3:**
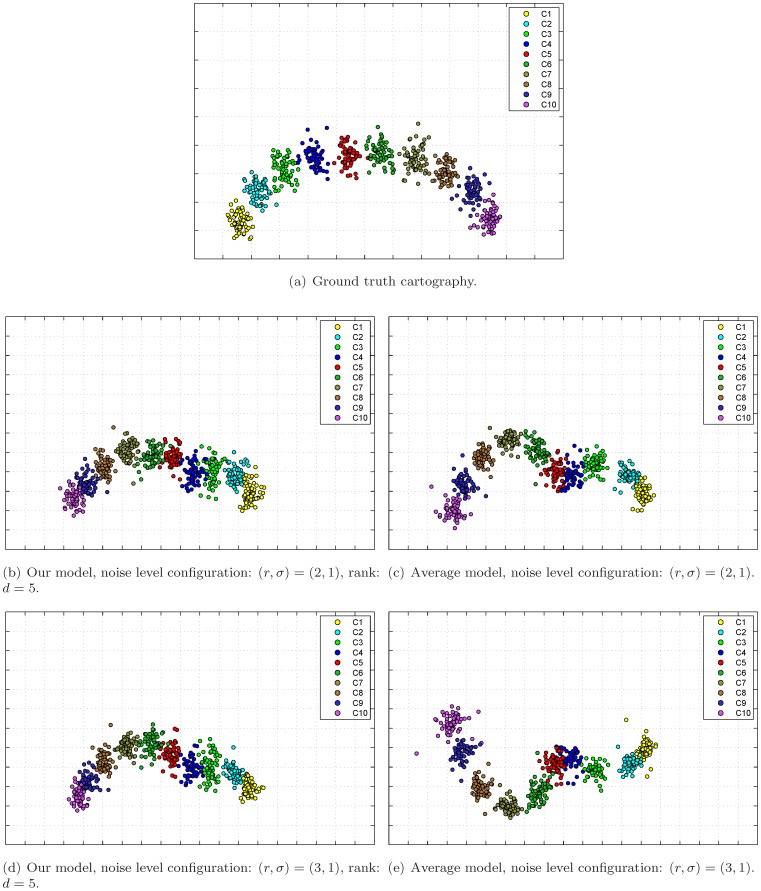
Antigenic cartographies constructed by the considered models on the simulated data. Here we use MC-MDS for cartography construction.

Figure 3 shows the constructed antigenic cartographies from the ground truth table, the integrated tables by our model as well as those by the average model. For each model, we show the cartographies constructed by MC-MDS [4] which is a representative MDS method for antigenic cartography construction. We show the results under noise level configuration 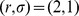 (see Figure 3(b)&3(c)) and 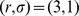 (see Figure 3(d)&3(e)). For each noise level configuration, we select the optimal rank 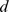 in our model based on the RMSE curves in Figure 1. Visual inspection on Figure 3 shows that the cartographies constructed from our model are closer to the ground truth than those constructed from the average model.

### Data Integration on HI Datasets of 2009 H1N1 Influenza A Viruses

**Figure 4:**
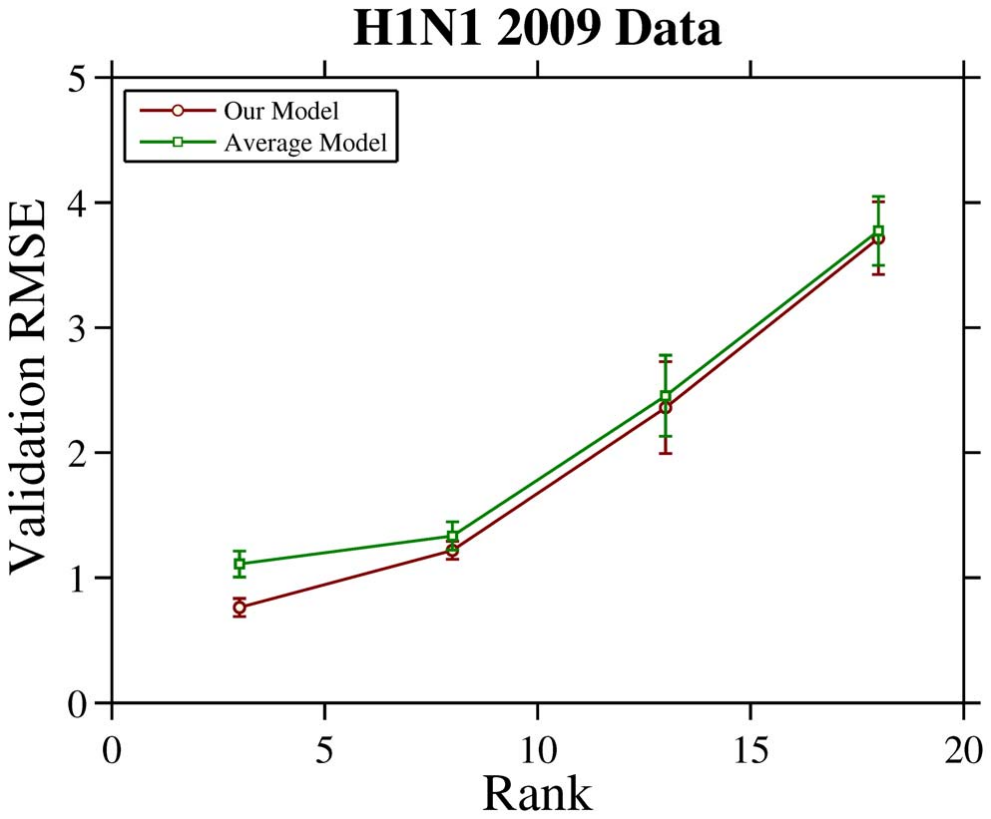
RMSE versus rank curves achieved by the considered models on H1N1 2009 data. The mean and deviation of RMSE are calculated by 10-fold cross validation.

The H1N1 2009 HI dataset contains 6 HI tables collected from May of 2009 to August of 2009. The merged table is of size 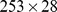, i.e., 253 viruses versus 28 antisera. The RMSE versus rank curves for H1N1 2009 are shown in Figure 4. The mean and deviation of RMSE are calculated by 10-fold cross validation. The curves show that our model consistently achieves lower RMSE than average model under different ranks, especially when rank is less than 10. This result supports the claim that explicitly modeling the antiserum bias across tables is effective to improve the prediction accuracy of table integration.

**Figure 5:**
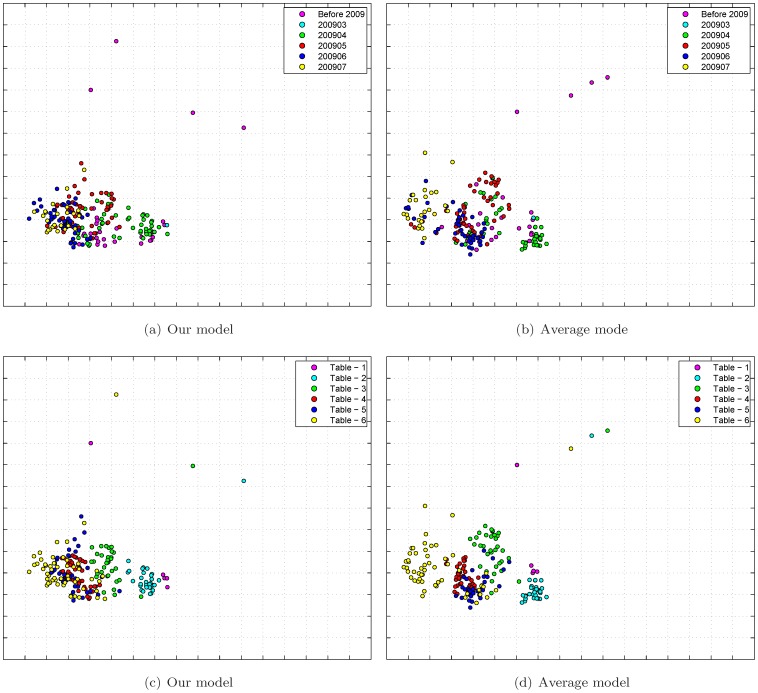
Antigenic cartography of H1N12009 dataset. Here the rank value 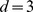 is selected in our model for matrix completion and MC-MDS is used for cartography construction. Top row: the antiviruses are colored by collected date. Bottom row: the antiviruses are colored by table.

Figure 5 shows the constructed antigenic cartographies from our model and the average model with MC-MDS. The scale of antigenic cartography is based on the antigenic distances from HI tables, e.g., each unit (grid) in the antigenic cartography represents a 2-fold change in HI titre. In Figure 5(a)&5(b), the antigens are colored by month in which they are collected, while the antigens are colored by tables in Figure 5(c)&5(d). Here we select rank 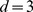 in our model through 10-fold cross-validation (see Figure 4).

The antigenic cartography by the average model showed that the viruses are separated by strips, indicating the viruses from different months are antigenically different. This is not true because there is no significant evidence demonstrating influenza antigenic drift in the first wave of 2009 H1N1 pandemic. In contrast, our joint matrix completion and filtering model was able to integrate these data, and the viruses from different months are mixed altogether. This is biologically sound.

### Data Integration on HI Datasets of 2000–2007 H3N2 Human Seasonal Influenza Viruses

**Figure 6:**
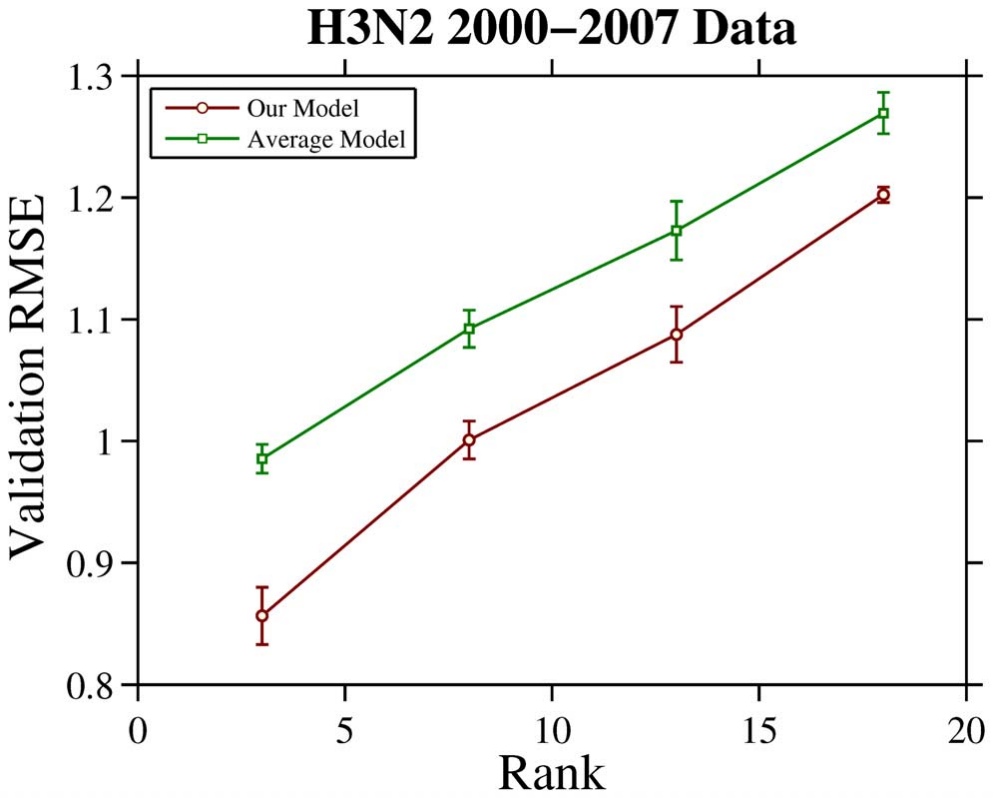
RMSE versus rank curves achieved by the considered models on H3N2 2000–2007 data. The mean and deviation of RMSE are calculated by 10-fold cross validation.

The H3N2 2000–2007 data used in our study contains a total of 369 individual HI tables performed from 2000 to 2007, including 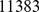 viruses and 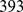 antisera. All HI experiments were performed using turkey red blood cells. The RMSE versus rank curves are plot in the Figure 6. The mean and deviation of RMSE are calculated by 10-fold cross validation. Once again, the curves suggest that our model outperforms average model in table integration accuracy.

**Figure 7:**
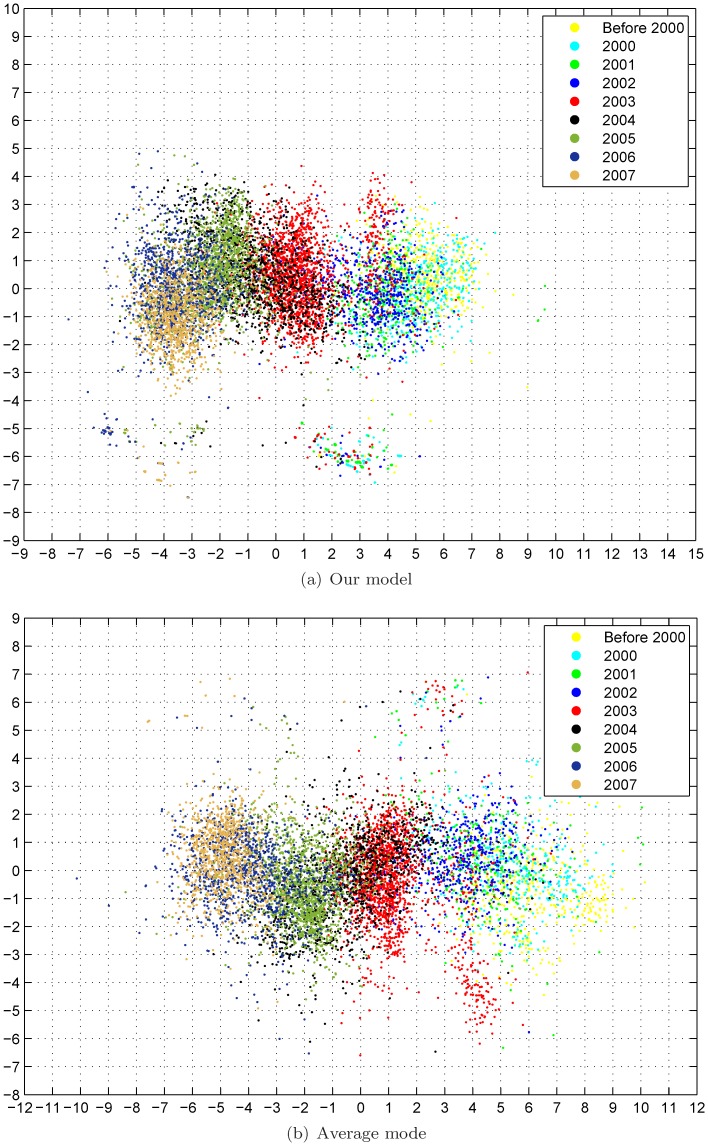
Antigenic cartography of H3N2 2000–2007 dataset. Here the rank value 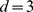 is selected in our model for matrix completion and MC-MDS is used for cartography construction.

Figure 7 shows the constructed antigenic cartographies for both our model and average model. These antigens are labeled according to their collected years. Visualization shows that the constructed cartography from our automatic model is more compact than that from average model. It is interesting to note that our cartography is consistent to that created from a manually curated HI table [11]. The Matlab code for reproducing the results in this paper will be available upon request.

## Discussion

In this work, we investigated the problem of combining a set of HI tables from different experiments in the same laboratories or even from different laboratories into an integrated table. With such a fusion of data, the rich but potentially redundant information contained in individual tables can be compactly and robustly represented in a single table which will in turn facilitate data visualization and higher level biological data mining tasks such as influenza surveillance. We note that each HI table (experiment) only includes a dozen of reference antisera, which are updated at each influenza season or even each month within the same influenza season. In addition, it is common for individual laboratories to use different antisera. Therefore, the integrated HI table is typically an incomplete matrix. For each pair of antigen-antiserum, its observed HI titre values in individual tables could be varying due to the random effects of antisera and the system noise. Therefore, the incompleteness and uncertainty of observations present two major challenges in combining tables into an integrated table.

We proposed a computational method for integrating multiple HI tables, and demonstrated its usefulness in antigenic characterization. We paid special attention to the major challenges caused by the incompleteness and the uncertainty of data. It is shown by extensive experiments that the standard approach by averaging the observations does not perform well. We proposed a simultaneous matrix completion and filtering model which carefully models different levels of random effects in each individual table. Our experimental results showed that more accurate results can be obtained through this new method. After table merging, we can use MDS to construct its antigenic cartography. In our experiments, we constructed antigenic cartography by using MC-MDS [4]. We have also tried another representative MDS method, the metric MDS [5], for cartography construction. The main difference between metric MDS and MC-MDS lies in that the former directly fits the HI titre (with proper transformation) of antigen-antiserum pairs while the latter regards each antigen as a row vector of data matrix and fits the pairwise Euclidean distances between antigens. Our unreported results suggest that both MDS methods work well although the MC-MDS tends to output more compact cartographies than metric MDS, as described elsewhere [4].

**Figure 8:**
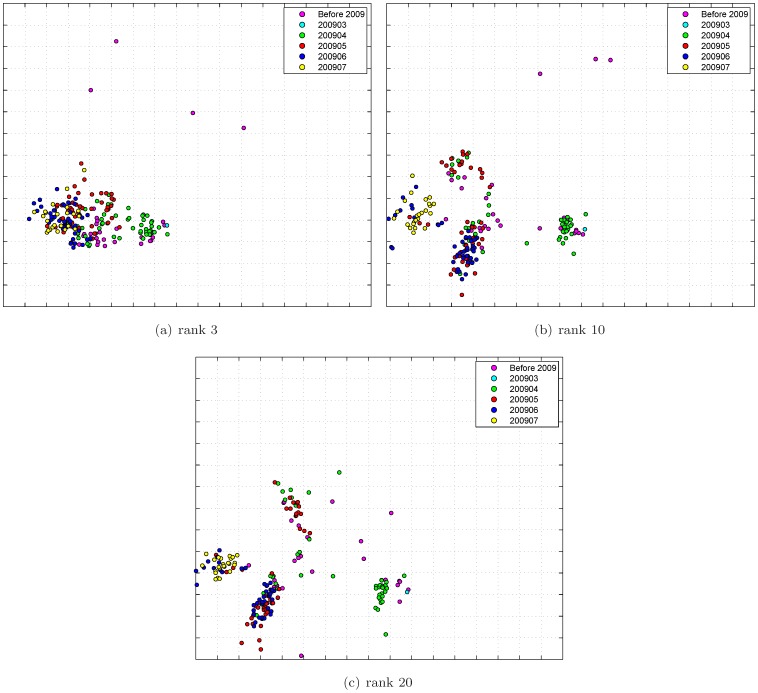
Antigenic cartography of H1N12009 data constructed by our model with different ranks.

As we have shown in Figure 4 and Figure 6 that the prediction performance deteriorates as the rank parameter of matrix completion increases. In fact, the same trend has also been observed in the constructed antigenic cartography. Figure 8 shows the antigenic cartographies of H1N1 2009 constructed by MC-MDS under rank 3, 10, 20. It can be seen that as rank increases, the antigenic cartography becomes more and more separative. This verifies that low rank assumption is vital for HI table integration.

The proposed data integration model can be used as a generic data integration model for biological datasets, especially the binding affinity data. The study of biological systems typically involves quantification of the binding affinity amongst different types of biological agents, which could be DNA, RNA, ligands, proteins, cells, or even living organisms, such as viruses. The reactions could be DNA versus DNA, DNA versus RNA, DNA versus protein, protein versus ligand (e.g. carbohydrates), and protein versus protein. Similar to HI data, the data point in these binding affinity datasets also suffers from missing values. For example, the missing data problem is also very common in high throughput DNA microarrays, protein microarrays and glycan microarrays, and these missing values can result from different version of array, poor printed spots in the slides, insufficient resolution, or hybridization problems [12]. Integration of this binding affinity data from individual experiments can be formulated to a similar problem as HI data integration studied in this paper. Thus, our proposed joint matrix completion and filtering model can be adapted as a general model for biological data integration, targeting data noises, missing values, and low reactors within and across experiments.

## Materials and Methods

### Generation of Simulated HI Datasets

In Monte Carlo simulation study, we generated a set of HI tables according to the data model (1). More specifically, we utilized the following steps to generate tables:

- Generate 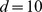 dimensional latent coordinates 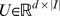 and 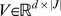 for 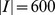 antigens and 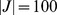 antisera, respectively. Both the antigens and antisera are equally partitioned into 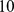 blocks. In order to generate such 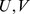, we first generate a matrix 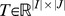 (the ground truth full table) with entry 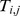 is randomly drawn from 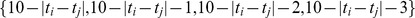 where 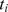, 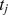 are the corresponding block index. We then calculate 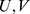 by rank-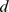 decomposition of 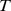 through singular value decomposition.
- Generate the antiserum bias 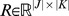 for 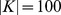 tables, with integer 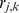 uniformly drawn from interval 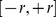. Choose variance 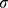 for the white Gaussian noise. We will consider different configurations of 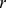 and 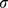 in our empirical study.
- Given 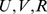 and 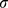, we randomly generate 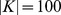 individual tables according to the linear model (1).

We generated 10 independent copies of the data and reported the mean and deviation of RMSE.

### 2009 H1N1 HI Dataset from Influenza Surveillance

**Figure 9:**
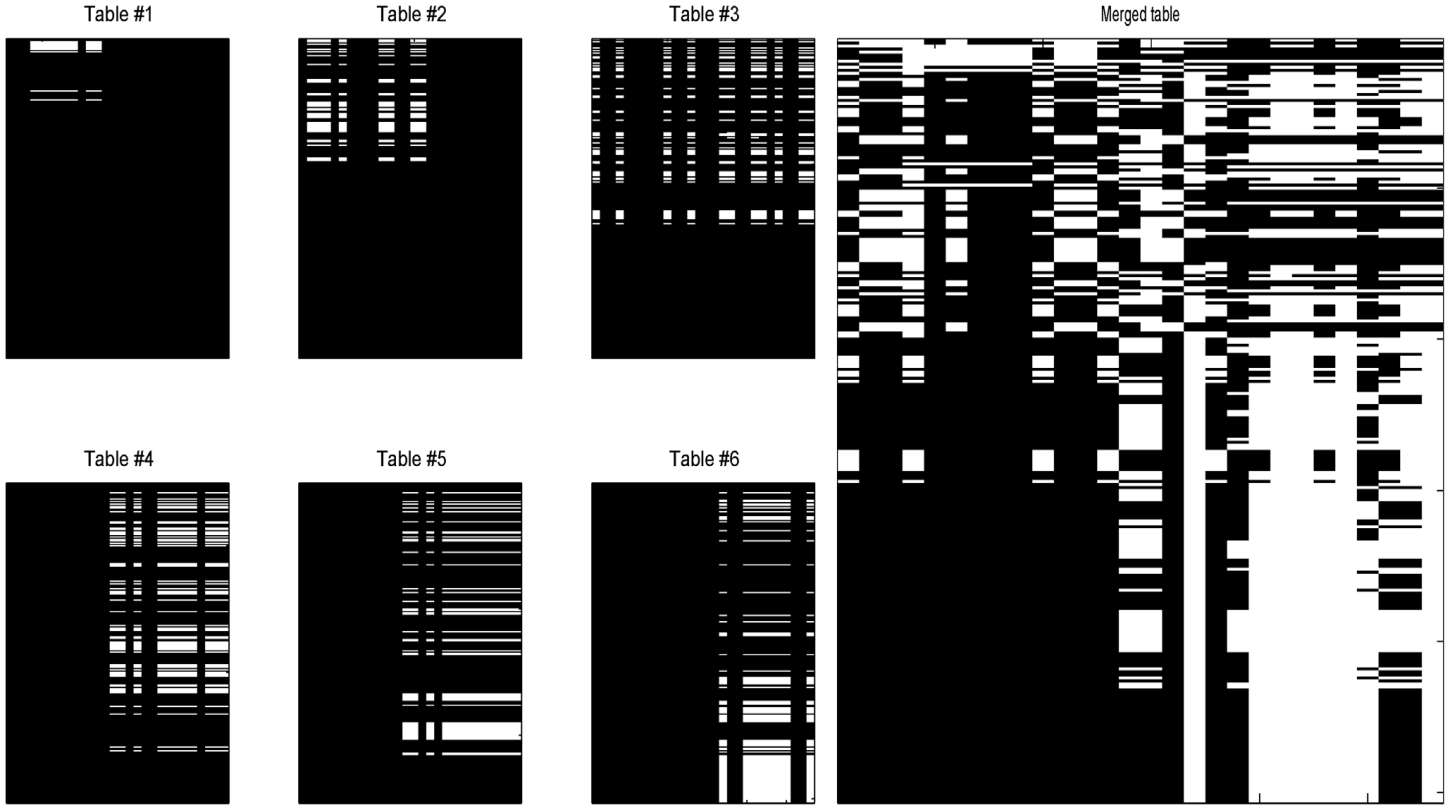
Data distribution in the H1N1 2009 dataset. Left panel: the individual tables; right panel: the merged table. White indicates entries observed, while black for missing entries. Both x-axis (index of antiserum) and y-axis (index of antigen) are temporally ordered.

The 2009 H1N1 HI dataset contains six individual HI tables spanning from April to August in 2009. The sizes of tables differ greatly, e.g., table #1 is of size 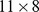, table #2 of 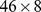 and table #6 of 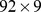. Each table only includes up to 16 reference ferret antisera, only some of them appear in two or more tables. A total number of 253 antigens (viruses) and 28 antisera are included from these six tables. Figure 9 shows the occurrence of the six HI tables in the merged 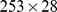 antigen-antiserum array (left panel), as well as the distribution of observed entries in the merged array (right panel). Data statistics show that there are only 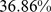 observed entries in the merged table.

Table 2 lists four selected antigen-antiserum pairs which have multiple observations of HI titre across the six HI tables in H1N1 2009. Note that one unit of HI titre represents 2-fold dilution of antiserum in the HI assay. It can be seen that the observed HI titre values are not consistent for 3 out of the 4 pairs. For example, there exists up to 4-fold divergence among the HI titers for the pair (A/New York/18/2009, A/California/7/2009). Therefore, uncertainty of observation presents another challenge for the table integration problem.

**Table 2:** H1N1 2009 dataset: examples of HI titre observations across multiple tables.

Antigen – Antiserum	T1	T2	T3	T4	T5	T6
A/Wisconsin/10/1998 –A/Wisconsin/10/1998	9	8	8	–	–	–
A/Brisbane/59/2007 –A/California/7/2009	–	–	0	0	0	1
A/New York/18/2009 –A/California/7/2009	–	–	8	7	9	7
A/Mexico/4108/2009 –A/TEXAS/15/2009	–	–	9	9	9	8

### 2000–2007 H3N2 Human Seasonal Influenza Viruses HI dataset

**Figure 10:**
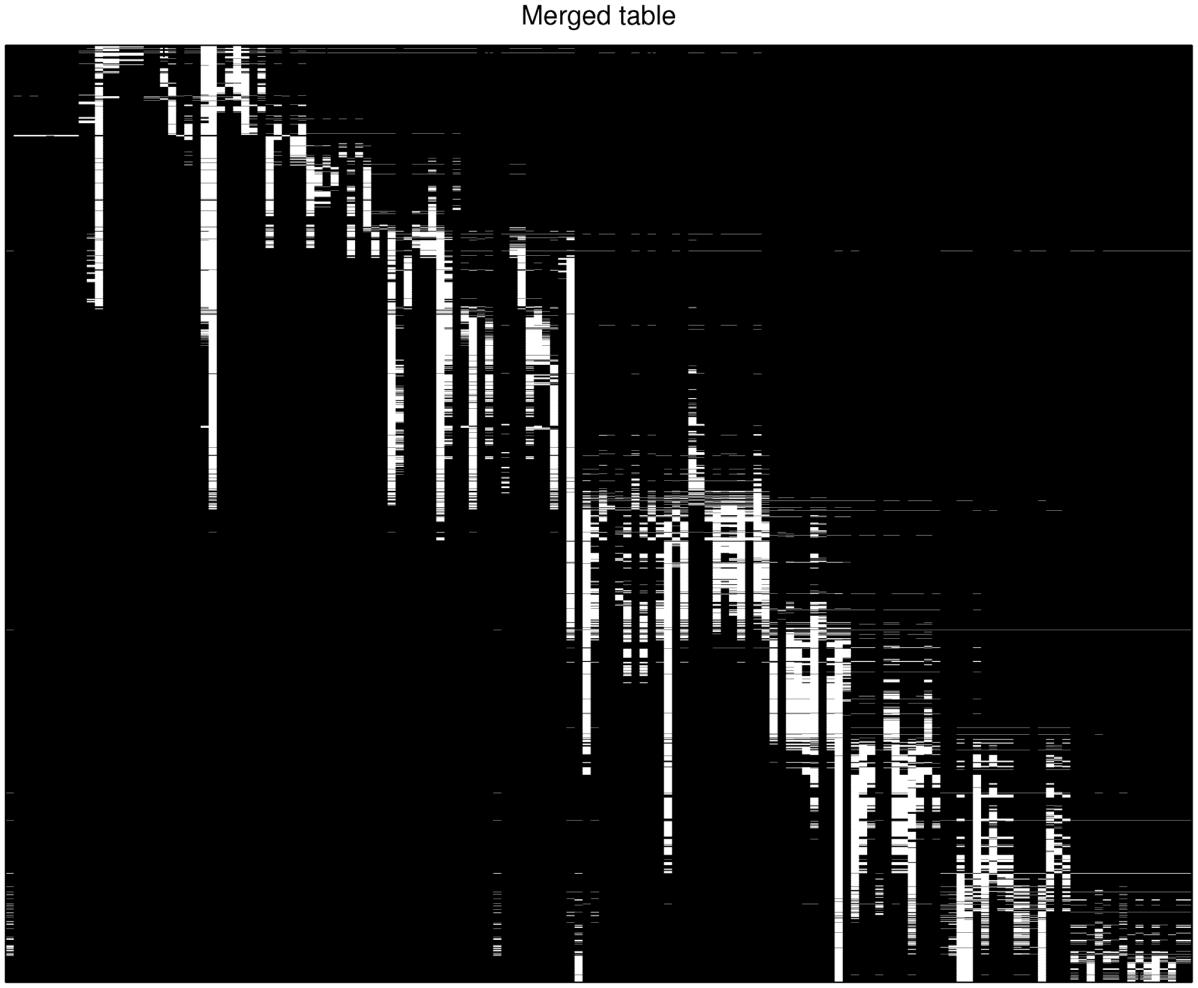
Data distribution in the H3N2 2000–2007 dataset.

In Figure 10, we show the distribution of observed entries in another dataset used in this study, the H3N2 2000–2007 datasets, in which the tables are collected from year 2000 to year 2007. This dataset contains 369 tables and the merged table has 11,383 antigens as rows and 393 antisera as columns. Visual inspection of Figure 10 shows that a large portion of the entries in the combined H3N2 table are missing, and there are only 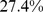 of observed entries. Similar to the H1N1 2009 dataset, the observed HI titre values of the same pair of antigen-antiserum in individual tables could be varying. One important task is to fill out the missing entries based on the noisy observations in individual tables.

### Performance Evaluation

In this study, the performance of our data integration model is evaluated using the following two criteria: root mean squared error and biological interpretation, which is mainly through antigenic cartography.

### Quantitative Evaluation by RMSE

Given 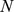 true values 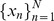 and prediction values 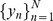, the root mean squared error is defined by:



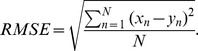
.

If a prediction scheme has a small RMSE value, then the predicted values are close to the true values. For real data, since we have no ground truth for the merged HI table, we calculate the RMSE values on individual tables through 10-fold cross-validation. The observed HI titre values 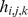 from the tables are partitioned into 10 equal parts. Each time, one part is used for testing and the remaining nine parts for training. That is, each time we estimate the parameters of model (2) using 9 parts as observed values, and then calculate RMSE between the predicted values and the observed values on the remaining part. The process is repeated for every part and we report the mean and standard deviation of RMSE. It is known that standard deviation calculation based on cross validation is often smaller than the true standard deviation; however, the numbers still provide meaningful indications and hence are included.

### Qualitative Evaluation by Antigenic Cartography

The biological interpretation is based on quantification of the reported antigenic variant groups in the influenza antigenic cartography. An antigenic cartography is a geometric representation of HI assay data. In such a cartography, the relative positions of antigens and antisera are adjusted such that the distances between antigens and antisera in the map represent the corresponding HI measurements with least error. Distance in the map thus represents antigenic distance and the closer antigens are to each other in the map the more similar they are antigenically.

Multidimensional scaling (MDS) is a statistical technique widely used in information visualization [13]. It attempts to embed a set of data into low dimensional vectors while preserving their pairwise distances. This technique has been applied to the construction of influenza antigenic cartography [4], [5], [14], [15]. Particularly, the MC-MDS method was developed in [4] to visualize antigenic distances. The goal is to minimize the error 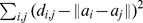 in which 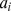 and 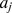 are two or three dimensional representation of antigen 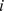 and antigen 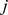. Here we conventionally define the antigenic distance 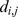 as the Euclidean distance between the row vectors associated with antigens 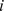 and 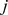 in the merged HI titre table. One unit of such an antigenic distance corresponds to a 2-fold dilution of antiserum in the HI assay.
